# Mechanistic basis for inhibition of the extended‐spectrum β‐lactamase GES‐1 by enmetazobactam and tazobactam

**DOI:** 10.1002/1873-3468.70155

**Published:** 2025-09-13

**Authors:** Michael Beer, Philip Hinchliffe, Marko Hanževački, Christopher R. Bethel, Catherine L. Tooke, Marc W. Van der Kamp, Krisztina M. Papp‐Wallace, Robert A. Bonomo, Stuart Shapiro, Adrian J. Mulholland, James Spencer

**Affiliations:** ^1^ School of Cellular and Molecular Medicine University of Bristol Bristol UK; ^2^ Centre for Computational Chemistry, School of Chemistry University of Bristol Bristol UK; ^3^ Research Service Louis Stokes Cleveland Department of Veterans Affairs Cleveland OH USA; ^4^ Department of Biology and Biochemistry 4 South, University of Bath Bath UK; ^5^ School of Biochemistry University of Bristol Bristol UK; ^6^ Department of Medicine Case Western Reserve University School of Medicine Cleveland OH USA; ^7^ Department of Biochemistry Case Western Reserve University School of Medicine Cleveland OH USA; ^8^ Department of Molecular Biology and Microbiology Case Western Reserve University School of Medicine Cleveland OH USA; ^9^ Department of Pharmacology Case Western Reserve University School of Medicine Cleveland OH USA; ^10^ CWRU‐Cleveland VAMC Center for Antimicrobial Resistance and Epidemiology (Case VA CARES) Cleveland OH USA; ^11^ Department of Proteomics and Bioinformatics Case Western Reserve University School of Medicine Cleveland OH USA; ^12^ Harry Lime Institute for Penicillin Research Basel Switzerland; ^13^ Present address: JMI Laboratories, a Subsidiary of Element Materials Technology North Liberty IA USA

**Keywords:** antibiotic resistance, β‐lactamases, β‐lactamase inhibitors, density functional theory, Py‐ChemShell, QM/MM

## Abstract

β‐Lactamase‐catalysed hydrolysis is the primary form of β‐lactam antibiotic resistance in Gram‐negative bacteria. The penicillanic acid sulfone (PAS) enmetazobactam is thought to inhibit extended‐spectrum β‐lactamases (ESBLs) by fragmentation of an initial acyl‐enzyme to form an active‐site lysinoalanine cross link. We investigate interactions of enmetazobactam and its congener tazobactam with GES‐1, an ESBL with structural features of carbapenem‐hydrolysing β‐lactamases. Crystal structures show different breakdown products of the two inhibitors covalently bound to the catalytic Ser70, assigned using quantum mechanics/molecular mechanics (QM/MM) calculations. We find no evidence for lysinoalanine formation, with mass spectrometry indicating active enzyme regeneration, behaviour previously observed for carbapenem‐hydrolysing enzymes, but not ESBLs. This work establishes that PAS inhibitors interact with diverse β‐lactamases by differing mechanisms, which should inform development of future compounds.

## Graphical abstract

Antimicrobial resistance (AMR) is of huge importance, resulting in over 1 million deaths each year. Here, we describe how a new drug, enmetazobactam, designed to help fight resistant bacterial diseases, inhibits a key enzyme (GES‐1) responsible for AMR. Our data show it is a more potent inhibitor than the related tazobactam, with high‐level computation describing its chemistry on binding.

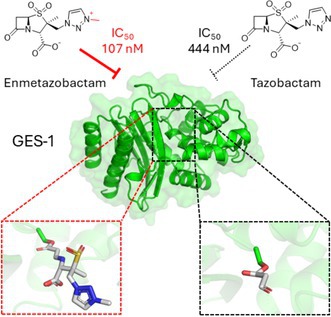

## Abbreviations

**2×YT**, 2× yeast extract tryptone

**BLI**, β‐lactamase inhibitor

**CPCM**, conductor‐like continuum polarisable model

**CTX‐M**, cefotaxime‐Munich

**cUTI**, complicated urinary tract infection

**DFT**, density functional theory

**ESBLs**, extended‐spectrum β‐lactamases

**GAFF**, general Amber forcefield

**GES**, Guiana extended‐spectrum

**IC**_**50**_, half‐maximal inhibitory concentration

**IPTG**, isopropyl β‐d‐1‐thiogalactopyranoside

**IRS**, inhibitor‐resistant SHV

**IRT**, inhibitor‐resistant TEM

**KPC**, *Klebsiella pneumoniae* carbapenemase

**MD**, molecular dynamics

**ND**, not determined

**OSBL**, original‐spectrum β‐lactamase

**PAS**, penicillanic acid sulfone

**PME**, particle mesh Ewald

**QM/MM**, quantum mechanics/molecular mechanics

**RSCC**, real‐space correlation coefficient

**SBLs**, serine β‐lactamases

**SHV**, sulfhydryl variable

**SME**, *Serratia marcescens* enzyme

**TEM**, Temoneira

**UPLC**, ultraperformance liquid chromatography

Loss of antibiotic efficacy due to selection of resistant mutants of target bacteria is a major and growing clinical concern. Overuse of antibiotics in the clinic and in agriculture has prompted rapid evolution of resistance in many pathogenic bacteria [1, 2]. Emergence and dissemination of β‐lactamases, that deactivate the most widely prescribed antibiotic class, β‐lactams [3, 4], represent a major hindrance to successful treatment of bacterial infections.

β‐Lactamases are divided into four groups according to Ambler [5]. Ambler classes A, C and D comprise the serine β‐lactamases (SBLs), which utilise an active site serine residue to catalyse hydrolysis of the β‐lactam ring, rendering β‐lactam antibiotics (and in some cases β‐lactamase inhibitors containing a β‐lactam moiety) inactive. Class A SBLs employ a catalytic Ser70 residue to hydrolyse β‐lactam antibiotics via a covalent acyl‐enzyme intermediate, with the acyl‐enzyme complex hydrolysed by nucleophilic attack of a water molecule (the deacylating water), activated by Glu166, to release an inactive cleavage product [6]. Class A enzymes have a spectrum of catalytic activity that collectively encompasses the full range of β‐lactam antibiotics [7].

The GES (Guiana extended‐spectrum) enzymes are a family of plasmid‐mediated class A β‐lactamases, currently comprising 67 variants [8], that are disseminated globally and are increasingly detected in *Pseudomonas aeruginosa, Acinetobacter baumannii* and *Klebsiella pneumoniae* [9, 10, 11, 12, 13]. The parent enzyme, GES‐1, is classified as an extended‐spectrum β‐lactamase (ESBL) due to an activity spectrum that encompasses penicillins and oxyimino‐cephalosporins (e.g. ceftriaxone, ceftazidime and cefepime), but not carbapenems [14, 15]; and its susceptibility to inhibition by the mechanism‐based β‐lactam inhibitor clavulanic acid (IC_50_ 5–7.7 μm [15, 16]). However, in contrast to other ESBLs, single point mutations, such as G170N (GES‐2) or G170S (GES‐5), endow detectable hydrolytic activity towards carbapenems, leading to carbapenem failure in patients infected by bacteria expressing GES‐2/GES‐5, or multiple other GES variants [17, 18, 19, 20].

A common strategy to treat infections by bacteria expressing β‐lactamases is to combine a β‐lactam with a β‐lactamase inhibitor (BLI) [6, 21, 22]. Clinically available BLIs act by forming a long‐lived covalent complex with the SBL catalytic serine (Ser70 in class A SBLs), functionally inactivating the SBL and preventing efficient hydrolysis of the β‐lactam antibiotic partner [6]. Clinically utilised BLIs are subdivided into three major scaffolds: β‐lactam based inhibitors (e.g. clavulanic acid, sulbactam and tazobactam) that are used most commonly; diazabicyclooctane inhibitors (e.g. avibactam); and boronate inhibitors (e.g. vaborbactam). Not unexpectedly, introduction of BLIs to the clinic has resulted in the emergence of β‐lactamases that are unaffected by BLIs, while maintaining activity against β‐lactam antibiotics [23].

**Fig. 1:**
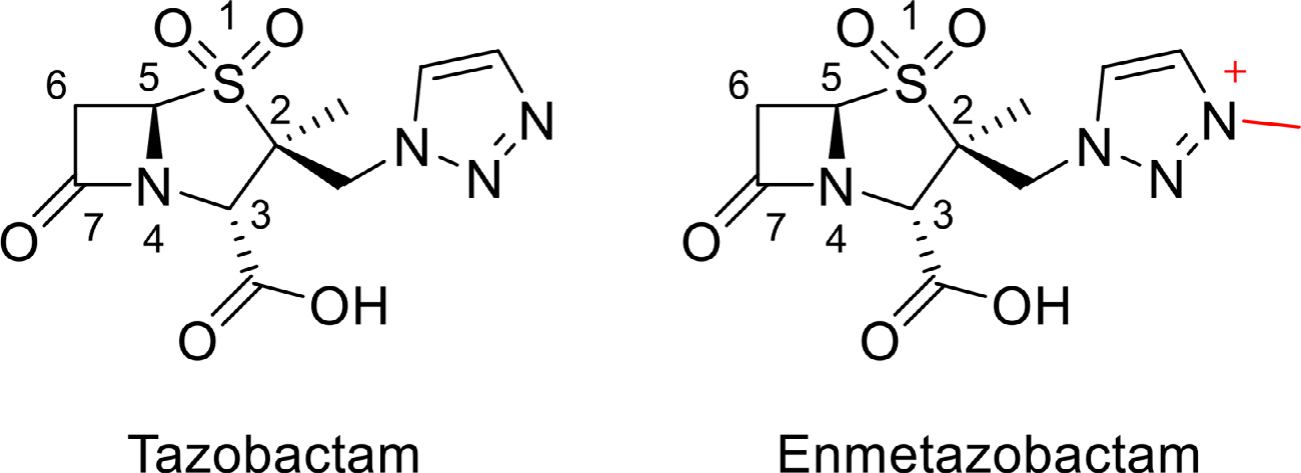
Structures of tazobactam and enmetazobactam. Enmetazobactam differs from tazobactam by the presence of a methyl group on the triazole ring, making it zwitterionic (red).

Enmetazobactam is a β‐lactam‐based BLI which has recently been approved for use in combination with cefepime by the European Medicines Agency (for complicated urinary tract infections (cUTI), hospital‐acquired and ventilator‐associated pneumonia and bacteremic sequelae thereof) [24] and the U.S. Food & Drug Administration (for cUTI) [25]. Enmetazobactam contains a penicillanic acid sulfone (PAS) scaffold [26, 27], like the older BLI tazobactam [28], but differs from the latter molecule by the strategic addition of a methyl group to the triazole ring (Fig. 1), rendering it zwitterionic [28] and accompanied by enhanced potency against bacterial cells [27]. It is postulated that the increased potency of enmetazobactam, compared to tazobactam, derives from its ability to penetrate the bacterial outer membrane and accumulate in the periplasm of susceptible pathogens at higher concentrations per unit time [27].

The reactions of PAS inhibitors with SBLs are complex (Scheme S1), initially involving the formation of an acyl‐enzyme, which ramifies into multiple intermediates, as observed by crystallography, mass spectrometry and Raman spectroscopy, when tazobactam or enmetazobactam is exposed to some SBLs: for example, class A (TEM‐1, TEM‐116, SHV‐1, CTX‐M‐9, CTX‐M‐15, CTX‐M‐64, GES‐2, and PC1), the class C (AmpC) enzyme CMY‐2, and the class D carbapenemase OXA‐48. These PAS inhibitor fragmentation pathways, after acylation and subsequent opening of the inhibitor thiazolidine ring, can result in an 88 Da hydrated aldehyde, a 70 Da aldehyde, and two separate 52 Da (vinyl ether and alkyne) adducts covalently bound to Ser70 (and to Ser130 in the case of the vinyl ether) (Fig. S1) [27, 29, 30, 31, 32, 33, 34, 35]. It is thought these different acyl‐adducts may have differing reactivities with respect to deacylation, as proposed for clavulanic acid [36]. Tazobactam and enmetazobactam behave similarly on reaction with the ESBL CTX‐M‐15, with the formation of a Ser70‐Lys73 lysinoalanine cross link as evidenced in co‐crystallisation and mass spectrometry experiments (Fig. S2) [27, 29, 37]. Mass spectrometry has shown that PAS binding to KPC‐2, a class A carbapenemase, is reversible, proceeding by the formation of a covalent complex upon exposure to either enmetazobactam or tazobactam, suggesting that class A carbapenemases are inhibited by enmetazobactam via covalent acyl‐enzyme formation, while in contrast class A ESBLs are inhibited by enmetazobactam via irreversible active site cross link formation [27, 29, 30, 38]. It is thought that differing conditions in mass spectrometry experiments favour specific fragmentation pathways, but little is known about how these fragmentation pathways may differ between proteins or whether their products are accessible by methods other than mass spectrometry [27, 29]. Understanding the difference between ESBL and carbapenemase inhibition by BLIs is important for the development of future agents that maximise efficacy across the full range of clinically relevant enzymes.

Here, we present kinetic, mass spectrometric, and crystallographic and molecular modelling data, characterising enmetazobactam‐ and tazobactam‐derived inhibitory complexes, that describe the reactions of the two PAS inhibitors with the class A β‐lactamase GES‐1. The results indicate that exposure of GES‐1 crystals to enmetazobactam leads to an initial acyl‐enzyme complex, most likely the *trans*‐enamine breakdown intermediate (Figs S1 and S3), whereas exposure to tazobactam results in a 70 Da aldehyde acylated to Ser70, rather than a ring‐opened acylated species. In contrast to earlier findings for ESBLs, formation of a lysinoalanine cross link between Lys73 and Ser70 was not observed after soaking GES‐1 with either PAS [37]. Our results uncover mechanistic details of class A β‐lactamase inhibition by enmetazobactam, which shows greater than 10‐fold more potent inhibition of multiple class A ESBLs than the related tazobactam. This work may aid in guiding future development of novel PAS BLIs.

## Methods

### Enzyme purification and crystallisation

The GES‐1 gene was synthesised with codon optimisation (Eurofins, Wolverhampton, UK). The putative signal sequence of the gene was removed by amplifying from position 52 using forward primer 5′‐AAGTTCTGTTTCAGGGCCCGGCGAGCGAGAAACTGACG‐3′ and reverse primer of 5′‐ATGGTCTAGAAAGCTTTATTTGTCGGTGGACAGGATCAG‐3′ before ligation‐independent insertion into the pOPIN‐F T7 expression vector [39], digested with KpnI (NEB, Ipswich, MA, USA) and HindIII (NEB). SoluBL21 (DE3) *Escherichia coli* (AMSBIO, San Diego, CA, USA) cells were transformed with the resulting pOPIN‐F‐GES‐1 plasmid and grown in 2× yeast extract tryptone (2×YT) media, supplemented with 50 μg·mL^−1^ of carbenicillin. Cells were shaken at 37 °C in 500 mL of liquid broth until an optical density at 600 nm (OD_600_) of 0.6–0.8 was achieved, at which time isopropyl β‐d‐1‐thiogalactopyranoside (IPTG) was added to a final concentration of 0.75 mm, and the cells left shaking at 18 °C overnight. Cells were harvested by centrifugation at 6500 ***g*** for 10 min at 4 °C and resuspended in 100 mL of buffer A (20 mm Tris pH 8.0, 300 mm NaCl) supplemented with one tablet of complete EDTA‐free protease inhibitor (Roche, Basel, Switzerland), 2 μL DNase I (NEB) and 2 mg lysozyme (Sigma, Burlington, MA, USA). Cells were lysed via one passage through a cell disruptor (Constant Systems, Daventry, UK) at a pressure of 25 kpsi, and pelleted by centrifugation at 100 000 ***g***, at 4 °C, for 1 h. The soluble fraction was incubated, in the presence of 10 mm imidazole, with 2 mL of Ni‐NTA resin (Qiagen, Venlo, the Netherlands) for 2 h at 4 °C. Beads were washed with 20 mL of 20 mm imidazole dissolved in buffer A and subsequently eluted with 10 mL of 300 mm imidazole dissolved in buffer B (20 mm Tris pH 8.0, 150 mm NaCl) at 4 °C. The eluent was concentrated and dialysed in an Amicon 10‐kDa molecular weight (Sigma) cut‐off centrifugal filter, at 4 °C, until the imidazole concentration reached 20 mm. Protein was then injected onto a HiLoad 16/600 Superdex 75 pg column (GE Healthcare, Amersham, UK), equilibrated with buffer B. Fractions (1 mL) were analysed using SDS/PAGE and those > 95% pure were pooled and concentrated as above to 30 mg·mL^−1^.

### Crystallographic data collection and structure determination

For ligand soaking experiments with enmetazobactam, purified GES‐1 was crystallised based on the conditions reported by Smith *et al*. [17]. Crystals were grown using sitting drop vapour diffusion in CrysChem24 plates (Hampton Research, Aliso Viejo, CA, USA) in which protein (5 mg·mL^−1^) was admixed 1 : 1 (4 μL final drop volume) with crystallisation mother liquor (10% (v/v) PEG 1000 and 10% (v/v) PEG 8000) and equilibrated against 500 μL mother liquor at room temperature. Crystals grew to maximum size (approx. 100 × 100 × 50 μm) within 3 weeks. Crystals were soaked in 30 mm of enmetazobactam (dissolved in mother liquor) for 1 min or for 2.5 h at room temperature. Therefore, crystals were soaked in a final 3 : 1 mixture of mother liquor and protein buffer (approximately 6.7 mm Tris pH 8.0, 50 mm NaCl, 6.7% (v/v) PEG 1000, 6.7% (v/v) PEG 8000 final concentrations) before brief (1–5 s) exposure to 25% (v/v) glycerol (4 μL drop volume) and cryo‐cooling in liquid nitrogen.

For tazobactam ligand soaking, a different crystallisation condition was required due to crystals grown in the above condition dissolving upon exposure to tazobactam. Therefore, purified GES‐1 (15 mg·mL^−1^) was added to a drop of 20 mm citric acid, 80 mm bis‐tris propane, 10% PEG 3350 at pH 8.8 in a 1 : 1 ratio. Crystals were then soaked in a solution of mother liquor supplemented with 10 mm of tazobactam for 2.5 h before brief exposure to 25% (v/v) glycerol and subsequent plunging into liquid nitrogen.

Diffraction data were collected at Diamond Light Source (Didcot, UK) on beamline I03 and indexed, integrated, scaled and merged using dials [40] in the xia2 [41] processing pipeline. Phases were solved using molecular replacement with phaser [42] in phenix [43], with uncomplexed GES‐1 (PDB ID 2QPN [44]) as the starting structure. Structures were completed with rounds of refinement in phenix and manual modelling in wincoot [45]. Ligand restraints were calculated using elbow in phenix [46]. Models were validated by molprobity [47] and phenix.

### Enzyme kinetics

Half maximal inhibitor concentration (IC_50_) values were obtained by following initial rates of nitrocefin (TOKU‐E, Sint‐Denijs‐Westrem, Belgium) hydrolysis by changes in absorbance at 486 nm [48]. The experiments were performed in kinetics buffer (10 mm HEPES, pH 7.5, 150 mm NaCl). Enmetazobactam and tazobactam (1 mm to 100 pm) were incubated with 10 nm GES‐1 for 10 min at room temperature before addition of nitrocefin to a final concentration of 100 μm. All reactions were followed at 25 °C in Greiner half‐area 96‐well plates in a CLARIOstar Plus microplate reader (BMG Labtech, Aylesbury, UK). IC_50_ values were calculated using graphpad prism 9 (GraphPad, La Jolla, CA, USA).

*K*_iapp_ and *k*_inact_/*K* were determined as previously described [27, 49, 50]. In short, 10 nm of enzyme was challenged with a 1 : 2 dilution series of inhibitor, starting from 1 mm final concentration, and 50 μm final concentration of nitrocefin. Hydrolysis was monitored through absorbance at 486 nm, initial rates were used for calculation of *K*_iapp_ and full hydrolysis curves for *k*_inact_/*K*.

*k*_off_ values were determined using the jump‐dilution method as previously described [27, 49, 50]. 1 μm of GES‐1 was incubated with 20 and 200 μm of enmetazobactam and tazobactam, respectively, for 15 min and 6 h. Incubations were then serially diluted using the jump‐dilution method (resulting in a final concentration of GES‐1 of 10 nm) and the hydrolysis of 50 μm nitrocefin was followed monitoring absorbance at 485 nm.

### Mass spectrometry

Mass spectrometry data were measured as previously described [37] for purified GES‐1 incubated for 15 min and for 24 h with either tazobactam or enmetazobactam. In brief, purified GES‐1 was subjected to electrospray ionisation‐mass spectrometry (ESI‐MS) after either enmetazobactam or tazobactam exposure (1 : 1 ratio) at *t* = 0, 15 min and 24 h. All reactions were quenched with 1% (v/v) acetonitrile and 0.1% (v/v) formic acid in water. After quenching, samples were injected into an Acquity H class ultraperformance liquid chromatography (UPLC) instrument equipped with a 1.7 μm 2.1 mm by 100 mm Acquity UPLC ethylene bridged hybrid C_18_ column (Waters) that had been equilibrated with 0.1% formic acid in water.

### QM/MM and QM modelling

Previous studies have shown that molecular simulations can assist crystallographic refinement [27, 37, 51]. To prepare systems for quantum mechanics/molecular mechanics (QM/MM) geometry refinements, explicit bulk solvent was added and relaxed around the crystallographic model. In this case, all crystallographically modelled atoms (all heavy atoms from the protein, ligand and crystallographically modelled waters) were restrained while bulk solvent was allowed to relax around them. Chain A of the 2.5 h enmetazobactam‐soaked GES‐1 crystal structure was used, keeping all crystallographic water molecules and one HEPES buffer molecule found near the protein‐bound enmetazobactam ligand. The protonation states of all titratable residues at pH 7.4 were calculated with propka 3.1 [52]. All residues were in the standard protonation states other than Lys234 and Asp245 (in the Ambler numbering scheme) which were deprotonated and protonated, respectively. Histidine tautomers were determined by the Reduce program implemented in ambertools21 [53]. The all‐atom AMBER ff14SB [54] force field was used to describe protein residues. After the addition of hydrogen atoms to protein‐bound enmetazobactam (both the enamine and imine tautomers were modelled) and HEPES (deprotonated form), both enmetazobactam and HEPES were parameterised using the General Amber Force Field (GAFF) [55] and their partial charges derived using the AM1‐BCC method with the Antechamber package [56]. The structure was solvated with a cubic box of TIP3P [57] water using the tleap module of AMBER 20 [58], such that the box edge was at least 10 Å from any solute atoms. No counterions were added because a neutral charge of the system was not required for the subsequent QM/MM or QM geometry optimisation calculations. All heavy atoms were initially restrained to their crystallographic positions with a force constant of 500 kcal·mol^−1^·Å^−2^. After an initial water energy minimisation (300 steps of steepest descent with 700 steps of conjugate gradient, with all nonwater heavy atoms restrained (500 kcal·mol^−1^·A^−2^ restraint)), and a second minimisation with only backbone atoms restrained (500 kcal·mol^−1^·A^−2^ restraint), the system was subject to MD simulation for 200 ps using the NVT ensemble, with restraints maintained on all nonwater heavy atoms. The system was heated to 298 K over the first 20 ps, with the temperature controlled using the Langevin thermostat with a collision frequency of 1 ps^−1^, followed by a further 5 ns of NPT simulation at a constant pressure of 1.0 bar using the Berendsen barostat, maintaining the restraints on all nonwater heavy atoms. All bonds involving hydrogens were constrained using the SHAKE algorithm. Long‐range electrostatic interactions were treated with the Particle Mesh Ewald (PME) method with a nonbonded cut‐off of 10 Å. A timestep of 2 fs was used in all molecular dynamics (MD) simulations, which were performed using the amber 20 software [58].

Final solvent equilibrated MD structures were processed to obtain nonperiodic systems suitable for QM/MM calculations, used to determine the dominant tautomeric form of the acylated enmetazobactam in the enmetazobactam‐derived GES‐1 acyl‐enzyme presented here. The solvation shell around enmetazobactam was created by retaining the closest 500 water molecules to the protein‐bound enmetazobactam ligand, while all others were removed. The active region for geometry optimisation (i.e. in which atoms were allowed to move during optimisation) was defined as all those residues whose atoms were within 6 Å of protein‐bound enmetazobactam, while the positions of all other atoms were fixed. The QM region was defined as acylated enmetazobactam, starting from Cβ of Ser70 (Fig. S4). The Cα of Ser70 was replaced by a single link hydrogen atom generated with covalent coupling. The QM region was comprised of 41 atoms, including the link hydrogen. The total charge and multiplicity of the QM region were 0 and 1, respectively. QM/MM geometry optimisation was performed with electrostatic embedding and additive scheme at the B3LYP/MM level (B3LYP/6‐31G(d):AMBER ff14ffSB) including Grimme's D3 dispersion correction and Becke–Johnson damping (D3BJ) [59, 60]. The QM/MM system was optimised to a minimum using the L‐BFGS algorithm with a constant trust radius. Single point calculations were performed on previously optimised geometries at the B3LYP‐D3(BJ)/def2‐TZVP level of theory. Frequency calculation was done at the geometry optimisation level of theory to confirm that the optimised structure is a true minimum. Free energy corrections at 300 K obtained from frequency calculations were included in the final electronic energies. All QM/MM calculations were performed using the ORCA 5.0.3/DL_POLY 5 interface in Py‐ChemShell 21.0.3 [61, 62].

Cluster models are a ‘QM‐only’ methodology where only some residues or parts of some residues (e.g. the Cα atom and the side chain) are modelled, reducing the computational cost by only considering a subset of atoms involved in a protein system. The active site cluster models used here were generated using the gaussview 6.1.1 package [63]: they included active site residue side chains (Ser70, Lys73, Asn132, Glu166, Thr237; Fig. S4) and the acylated enmetazobactam in either the *trans*‐enamine or imine forms. Solvent and buffer molecules were not included in the active site model (Fig. S4). Geometry optimisations were performed while restraining atoms as shown in Fig. S4 to maintain the positions of active site residues and acylated enmetazobactam close to the crystallographic conformation. Geometry optimisations were performed using the implicit solvation (conductor‐like continuum polarisable model (CPCM), ε = 4.24) at the density functional theory (DFT) level using the B3LYP functional and 6‐31G(d) basis with Grimme's D3 dispersion correction (important for modelling structures and interactions in proteins [64]) and Becke–Johnson damping, set in the gaussian 16 package [65]. Single point energies were calculated using the B3LYP functional and a larger (def2‐TZVP) basis set including Grimme's D3 dispersion correction and Becke–Johnson damping. QM calculations of isolated acylated enmetazobactam (including Ser70 side chain) also were performed at the same DFT levels: the B3LYP/6‐31G(d) level of theory was used for geometry optimisation, and the B3LYP/def2‐TZVP method (both calculations included Grimme's D3 dispersion correction and Becke–Johnson damping) for single point energy calculations.

## Results

### Enmetazobactam is a more potent inhibitor of GES‐1 than tazobactam

Enmetazobactam shows different inhibitory potencies towards different class A β‐lactamases, with notably lower IC_50_s for ESBLs than for carbapenemases (Table 1). With a few exceptions, reported IC_50_ values for enmetazobactam are lower than those for tazobactam for a diverse panel of class A β‐lactamases. The IC_50_ of enmetazobactam for purified GES‐1 is 107 nm, fourfold lower than that of tazobactam (444 nm), signalling that enmetazobactam is a more potent inhibitor of GES‐1 than tazobactam. However, both tazobactam and enmetazobactam are less potent inhibitors (up to 10‐fold increase in IC_50_ values) of GES‐1 than of the original‐spectrum β‐lactamases (OSBLs) and ESBLs (Table 1) [27, 29, 30, 66]. Indeed, the potency of tazobactam against GES‐1 is similar to that towards inhibitor‐resistant variants of other ESBLs, for example, TEM‐30, SHV‐49 and SHV‐1^R244S^. These differences may be, in part, due to differing conditions of the individual kinetic experiments, although all were carried out at similar pH values.

**Table 1:** IC_50_ values of enmetazobactam and tazobactam against class A β‐lactamases. CTX‐M, cefotaxime‐Munich; ESBL, extended‐spectrum β‐lactamase; GES, Guiana extended‐spectrum; IRS, inhibitor‐resistant SHV; IRT, inhibitor‐resistant TEM; KPC, *Klebsiella pneumoniae* carbapenemase; ND, not determined; OSBL, original‐spectrum β‐lactamase; SHV, sulfhydryl variable; SME, *Serratia marcescens* enzyme; TEM, Temoneira.

Enzyme	Enzyme classification	Enmetazobactam IC_50_ (nm)	Tazobactam IC_50_ (nm)	References
GES‐1	ESBL	**107**	**444**	This Work
CTX‐M‐14	ESBL	6	100	[27]
CTX‐M‐15	ESBL	7	6	[27]
TEM‐26	ESBL	82	23	[27]
TEM‐1	OSBL	7	30	[27]
TEM‐116	OSBL	36	11	[29]
SHV‐1	OSBL	8	26	[27]
GES‐2	Carbapenemase	ND	500	[30]
SME‐1	Carbapenemase	ND	160	[66]
KPC‐2	Carbapenemase	360	3700	[27]
KPC‐3	Carbapenemase	520	4400	[27]
TEM‐30	IRT	290	240	[27]
SHV‐1^R244S^	IRS	560	390	[27]
SHV‐49	IRS	360	730	[27]

Bold values indicate results from the current study.

IC_50_ values for covalent enzyme inhibitors frequently show time dependence [67], as previously demonstrated for PAS inhibition of other β‐lactamases [27, 29]. Accordingly, to assess the effect of incubation time on inhibition, we also determined IC_50_ values for inhibition of GES‐1 by the two PAS compounds after 1 h of pre‐incubation, as opposed to 10 min (Table S3). These show ~ 4‐ and 2‐fold decreases in the IC_50_ values for both tazobactam and enmetazobactam, with enmetazobactam the more potent inhibitor at both time points (Table S3). The apparent inhibition constant (*K*_iapp_) for both enmetazobactam and tazobactam inhibition of GES‐1 was also determined (Table S1, Fig. S5). As expected, enmetazobactam has moderately greater potency than tazobactam, (*K*_iapp_ values 0.48 and 0.55 μm for enmetazobactam and tazobactam, respectively). Calculation of the second order rate constant for inactivation *k*_inact_/*K* revealed that enmetazobactam acylates GES‐1 at a rate over 100‐fold greater than does tazobactam (8400 m ^−1^·s^−1^ compared to 51 m ^−1^·s^−1^, Table S1, Fig. S5). This is consistent with the IC_50_ values, which show a more significant increase in potency for tazobactam (fourfold) than enmetazobactam (twofold) after 1 h pre‐incubation. The turnover rate of enmetazobactam by GES‐1, as determined by calculation of the rate constant for dissociation, *k*_off_, is also slightly greater than that for tazobactam (Table S2, Fig. S6), resulting in half‐lives for inhibition (*t*_1/2_ values) of 2.0 and 8.8 min for enmetazobactam and tazobactam, respectively.

**Fig. 2:**
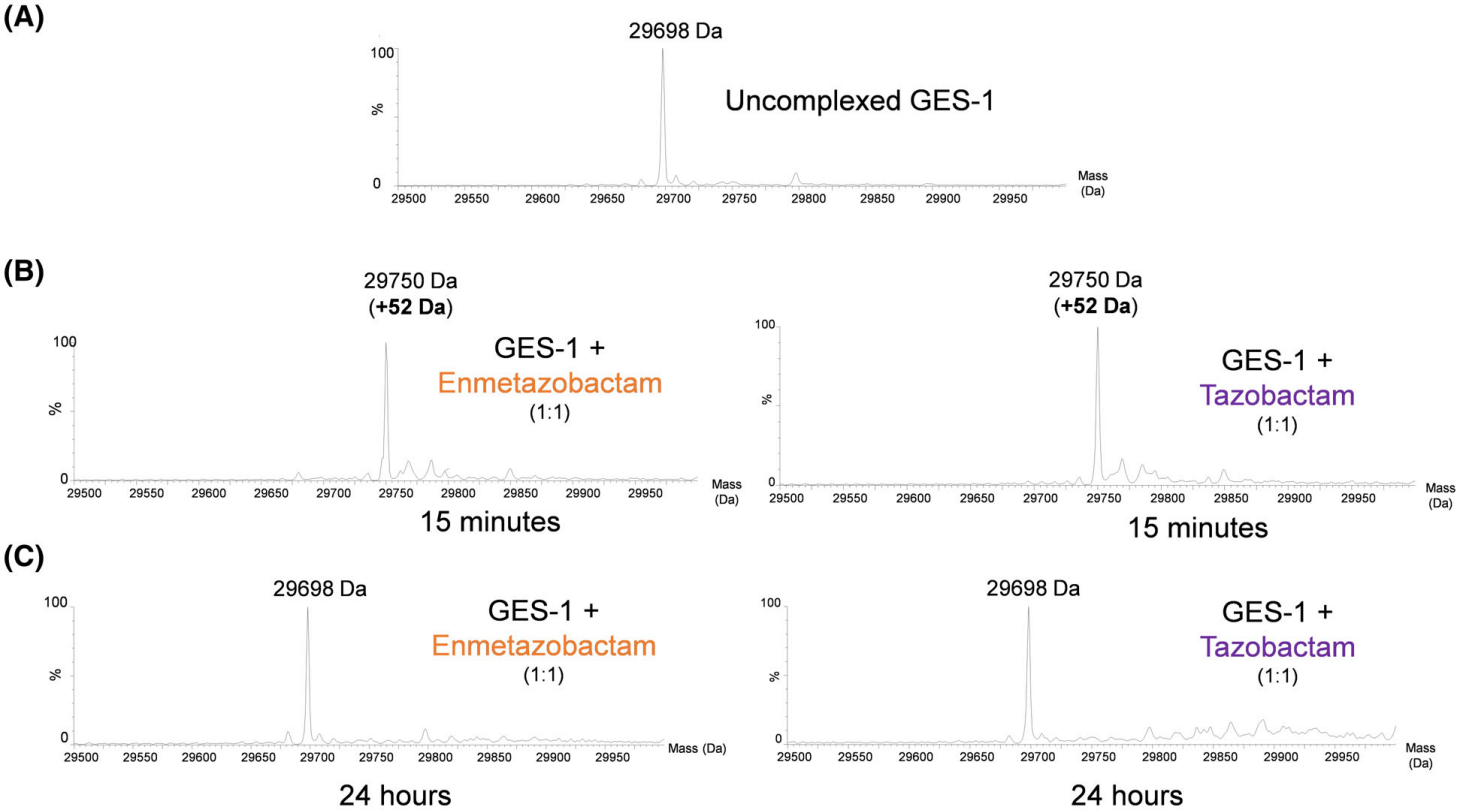
Mass spectrometric analysis of Guiana extended‐spectrum (GES)‐1 after exposure to enmetazobactam. (A) Mass spectrum of the native GES‐1 enzyme, in the absence of enmetazobactam exposure. (B, C) Mass spectra of GES‐1 following 15 min and 24 h exposure to enmetazobactam and tazobactam, respectively.

**Fig. 3:**
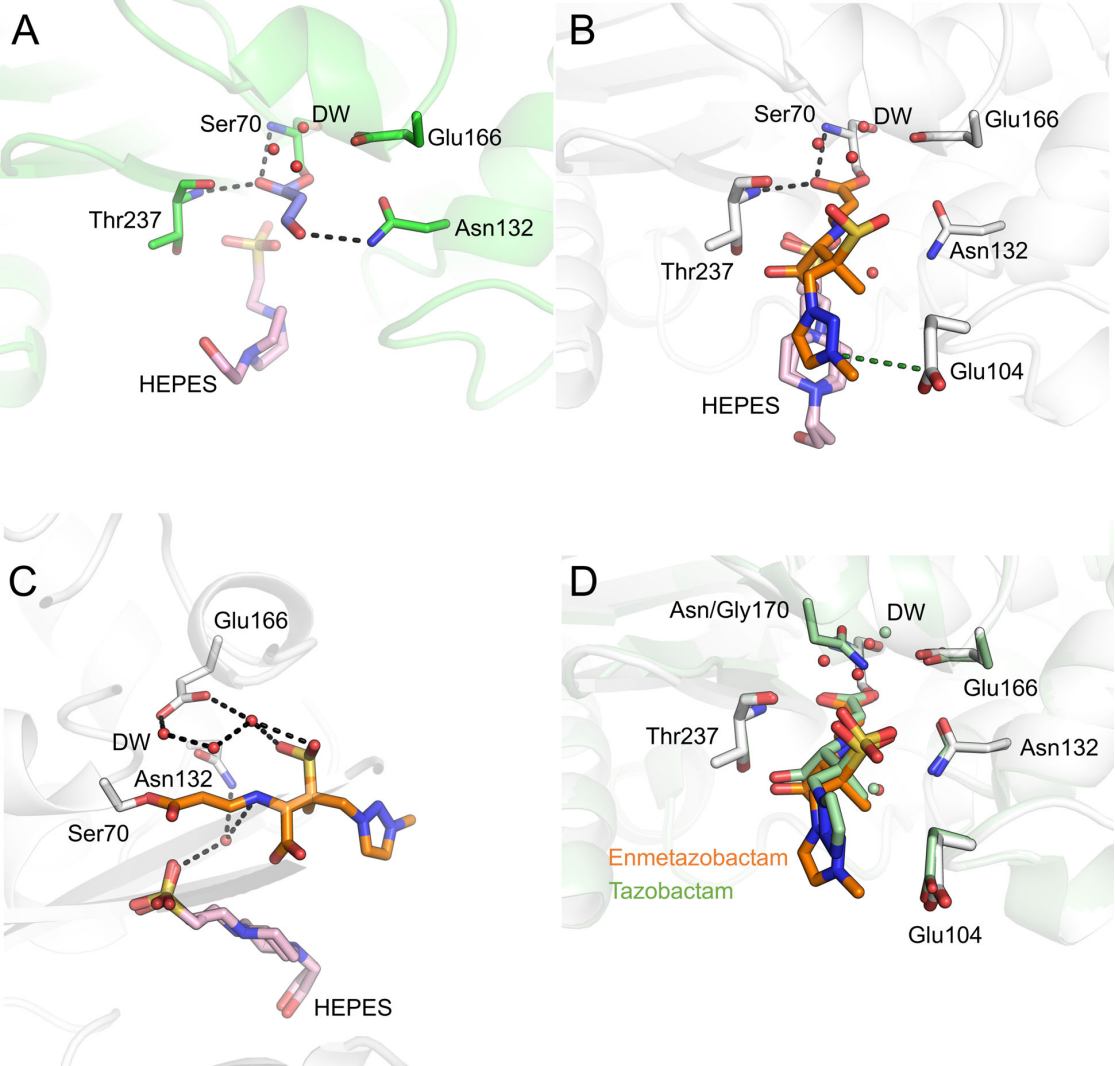
Crystallographic observation of tazobactam‐ and enmetazobactam‐derived Guiana extended‐spectrum (GES)‐1 acyl‐enzymes. (A) Tazobactam‐derived GES‐1 acyl‐enzyme. The 70 Da covalent adduct has been modelled into the electron density (Figs S1 and S7). Hydrogen bonds between the tazobactam‐derived covalent adduct and GES‐1, including bridging interactions with water molecules, are shown (black dashes). (B) Enmetazobactam‐derived GES‐1 acyl‐enzyme. The *trans*‐enamine tautomer was modelled into *F*_o_‐*F*_c_ electron density, as supported by quantum mechanics/molecular mechanics (QM/MM) structural modelling at the density functional theory (DFT) level of theory. Hydrogen bonds between the enmetazobactam‐derived covalent adduct and GES‐1 are shown (black dashes) along with the electrostatic interaction between Glu104 and the positively charged methyltriazole ring (green dashes). (C) A network of water molecules is resolved around the enmetazobactam‐derived GES‐1 acyl‐enzyme. Hydrogen bonds are highlighted with black dashed lines. (D) Overlay of tazobactam‐derived GES‐2 acyl‐enzyme (green protein and ligand, PDB ID 3NIA  [30]) and the enmetazobactam‐derived GES‐1 acyl‐enzyme presented here (grey protein, orange ligand). GES‐2 is a Gly170Asn mutant of GES‐1, and the addition of the side‐chain amide in GES‐2 results in sufficient electrostatic interactions to positionally stabilise a water molecule in the deacylating water (DW) position in GES‐2 crystal structures. As such, a DW is also observed in the tazobactam‐derived GES‐2 acyl‐enzyme (PDB ID 3NIA [30]), but is positioned 1 Å away from the putative DW in the enmetazobactam GES‐1 acyl‐enzyme, probably as a result of steric constraints from the Gly170Asn substitution in GES‐2. Images in the figure were generated using Schrödinger pymol 3.0.0.

We note that the IC_50_ values reported here are lower than the calculated values for *k*_off_/(*k*_inact_/*K*_I_) (Table S2). This is unusual among studies of PAS inhibition of β‐lactamases, where IC_50_ values are generally higher than those for *k*_off_/(*k*_inact_/*K*_I_), but is not unprecedented (e.g., see data for OXA‐10 in Lang *et al*. [29] and OXA‐48 in Vallejo *et al*. [68]). These discrepancies identify that the widely used kinetic treatment applied here represents a limitation of this, and other, studies of serine β‐lactamase inhibition by PAS and other β‐lactam‐derived inhibitors that undergo complex fragmentation pathways. Investigations of the dependence of *k*_off_ on the duration of pre‐incubation reveal little difference in the calculated values, with those for enmetazobactam unchanged within error, and those for tazobactam increasing by ca. threefold (Table S2). Such small differences also likely reflect the heterogeneity of the population of the PAS‐derived acyl‐enzyme, and derived products, that is expected to increase at longer pre‐incubation times (Figs 2 and 3). Furthermore, the *k*_off_ values indicate that both tested PAS inhibitors are deacylated and released from the enzyme active site relatively rapidly, in comparison to other β‐lactamase:BLI combinations, such as KPC‐2 with cyclic boronates, or avibactam, or relebactam with *Stenotrophomonas maltophillia* L2 or CTX‐M‐15 [27, 49, 50]. Nevertheless, the *k*_off_ and *t*_1/2_ values for GES‐1 inhibition by enmetazobactam and tazobactam determined here are similar to those previously reported for inhibition of other enzymes: CTX‐M‐15, TEM‐116, KPC‐2 and OXA‐10 [27, 29].

### GES‐1 exposure to penicillanic acid sulfones does not lead to the formation of a lysinoalanine cross link

To understand the modes of inhibition of GES‐1 by PAS compounds, the purified enzyme was exposed to tazobactam and enmetazobactam and the resultant covalent products (after 15 min and 24 h incubation) analysed using mass spectrometry. Mass spectra obtained following incubation with enmetazobactam or tazobactam are very similar. At 15 min, there is a single major peak (+52 Da), indicative of the vinyl ether product observed previously by Papp‐Wallace *et al*. and by Lang *et al*. (Fig. S1) [27, 29]. At 24 h, a single major peak at the mass of uncomplexed GES‐1 indicates regeneration of the native enzyme, most likely after deacylation of the acyl‐enzyme complex. Contrary to observations for CTX‐M‐15 (ESBL) and OXA‐10 (class D oxacillinase) [29, 37], an 18 Da mass loss corresponding to the loss of the Ser70 hydroxyl group, and formation of the Ser70‐Lys73 lysinoalanine cross link (Fig. 2, Fig. S2), was not observed.

### Crystallographic analysis of penicillanic acid sulfone reactions with GES‐1

To visualise the products of the reaction of enmetazobactam and tazobactam with purified GES‐1 using crystallography, GES‐1 crystals were incubated with 30 mm enmetazobactam or 10 mm tazobactam for 2.5 h. Crystallographic data (collected at cryogenic temperature) extended to 1.23 and 1.36 Å resolution for the enmetazobactam and tazobactam soaks (Table S4). Both crystal forms contained two molecules in the crystallographic asymmetric unit (chains A and B, respectively), as observed in GES variants previously crystallised in the same conditions [44, 69]. Size exclusion chromatography indicates that this is a nonphysiological dimer, forming during crystallisation. We do not observe, in any of the structures presented here, electron density in the active site that could be attributed to the 52 Da vinyl ether resolved by mass spectrometry (Fig. 2, Fig. S1). This matches previous reports of differences between crystallographically resolved structures and dominant adducts in solution identified by mass spectrometry [27, 29, 37], which may result from differing buffer conditions between mass spectrometric measurements and crystallographic ligand soaking. Indeed, the 52 Da adduct was noted to be promoted by the acidic conditions in the mass spectrometry experiment [29].

Active site electron density after GES‐1 exposure to tazobactam was modelled as the 70 Da aldehyde (Fig. 3, Figs S1 and S7) covalently bound to Ser70. The 70 Da aldehyde covalent adduct is thought to form after fragmentation of the initial acylated inhibitor [27, 29]. Lang *et al*. postulated that formation of the aldehyde is acid‐promoted, but the conditions used here to obtain the crystal structure of the tazobactam‐derived acyl‐enzyme were at pH 8.8, indicating that other factors may also promote the reaction pathway for aldehyde formation. As is common for β‐lactamase complexes with covalently bound β‐lactam antibiotics, or classical BLIs, the C7 oxygen (Fig. 1) is hydrogen‐bonded by the backbone amides of Thr237 and Ser70 (Fig. S8). A further hydrogen‐bonding interaction occurs between the aldehyde oxygen and the amide nitrogen of Asn132 (Fig. S8). No further interactions between the covalent adduct and protein were identified. Three water molecules are situated between Glu166 and the acylated aldehyde, hinting at their potential involvement in a deacylation reaction to regenerate the uncomplexed GES‐1 enzyme, as observed by mass spectrometry (Figs 2 and 3). A low occupancy HEPES molecule, hydrogen‐bonded to Ser130, was also resolved in the active site (Fig. S9). *F*_o_‐*F*_c_ electron density at a packing interface could be further modelled as a molecule of intact tazobactam (Fig. S10).

X‐ray diffraction data collected from GES‐1 crystals after exposure to enmetazobactam revealed electron density in the GES‐1 active site consistent with the imine or *trans‐*enamine tautomer of the ring‐opened acyl‐enzyme product (Figs S1, S3, and S11). Modelling of the *trans*‐enamine tautomer followed the observation that the linear difference electron density more closely matched a planar *trans*‐enamine conformation than an imine. The acyl‐enzyme product is thought to form early in the reaction pathway of PAS compounds with class A SBLs and has been documented in structures of SBL complexes with PAS inhibitor‐derived species [27, 28, 29, 30, 37]. No electron density consistent with the formation of the 52 Da vinyl ether, detected by mass spectrometry (Fig. 2, Fig. S1) and expected to be bound to both Ser70 and Ser130, was observed. Thus, the pathway for vinyl ether formation may be promoted by the conditions of mass spectrometry experiments, a conclusion reached previously about other breakdown fragments [29]; while, in the case of enmetazobactam, the acylated sulphone may be stabilised by conditions within the crystal.

The enmetazobactam‐derived acyl‐enzyme forms only a few electrostatic interactions with active site residues of GES‐1 (Fig. S8). The C7 oxygen (Fig. 1) sits within the oxyanion hole (formed by the backbone amides of Ser70 and Thr237, Fig. 3), consistent with structures of other β‐lactam antibiotics and BLIs covalently bound to class A β‐lactamases [6]. No other enzyme:enmetazobactam hydrogen bonds are formed in the acyl‐enzyme complex. Similarly, few enzyme:PAS inhibitor hydrogen bonds were observed in the crystal structure of the GES‐1^G170N^ variant, GES‐2, in complex with tazobactam‐derived inhibitory species [30]. However, a number of water molecules in the GES‐1:enmetazobactam complex structures presented here appear to make bridging interactions connecting the acylated enmetazobactam moiety and enzyme active site residues (Fig. 3B,C), including between the enmetazobactam sulfone and the side chain oxygen of Glu166, and with the backbone carbonyl of Gly170. Furthermore, the positive charge around the triazole ring (conferred by the additional methyl group of enmetazobactam compared to tazobactam; Fig. 1) forms a weak electrostatic interaction with the negatively charged side chain of Glu104 (distances between the positively charged nitrogen of the enmetazobactam triazole ring and Cδ of Glu104 4.3 and 3.8 Å, in chains A and B, respectively, Fig. 3).

An additional noteworthy observation in the crystal structures presented here, compared to other GES‐1 structures, is the presence of a water molecule in the deacylating position [6] (Fig. 3). Such a water molecule has not been observed previously in crystal structures of GES‐1, probably due to the inability of Gly170 (a position commonly occupied by Asn in other class A β‐lactamases, including those listed in Table 1) to participate in side‐chain hydrogen bonds [17, 44]. This water molecule is surrounded by a further network of water molecules, also not observed in other GES‐1 structures, that includes a water bridging the sulphone group of the enmetazobactam‐derived acyl‐adduct and the putative deacylating water. The observation of a putative deacylating water is consistent with mass spectrometry results that show GES‐1 as capable of turning over the species formed on reaction with enmetazobactam. The putative deacylating water is in an optimal position for proton transfer to Glu166 (or possibly Lys73) and to make a nucleophilic attack upon the C7 carbon (Fig. 1) of Ser70‐bound species in the first step of the deacylation reaction.

In both the tazobactam‐ and enmetazobactam‐derived complexes, further electron density in the active site could be modelled as a molecule of HEPES (present during GES‐1 purification). In the enmetazobactam‐derived complex, this HEPES molecule was modelled in dual conformation (Fig. S9). Electron density in the same position was also observed in the presented structure of otherwise uncomplexed GES‐1, supporting the decision to model this as HEPES (Fig. S9, Table S4). This density is in a similar position to the noncovalent product modelled in the previously reported GES‐2:tazobactam complex structure [30], but superposition of the respective electron density maps and structures does not suggest that HEPES would be a better fit in the GES‐2:tazobactam complex structure, or that the previously described noncovalent product would be a better fit to the current data (Fig. S12 [30]).

### QM/MM calculations indicate that the *trans‐*enamine is the dominant tautomer in GES‐1:enmetazobactam complexes

Due to resolution limits and the inherent complications of modelling mixed populations of tautomers, the crystal structures of class A β‐lactamase:(enme)tazobactam complexes presented here, and those previously deposited, alone have insufficient resolution to determine whether the favoured acyl‐enzyme tautomeric state is the *trans*‐enamine or the imine [37]. Molecular dynamics simulations of CTX‐M‐15:tazobactam complexes, and previous analyses of dihedral angles of SBL:tazobactam complexes [27], support the imine as the favoured form. However, other crystallographic and spectroscopic studies suggest that, while there may be a mixed population of both tautomers, the *trans*‐enamine is the dominant species [30, 32, 70, 71, 72, 73, 74, 75]. Here, modelling either the imine or *trans*‐enamine species into experimental electron density gave similar real‐space correlation coefficient (RSCC) values: 0.94/0.95 (chains A/B) for the *trans*‐enamine and 0.94/0.94 for the imine, respectively, and therefore does not distinguish between them. To resolve this uncertainty, QM/MM calculations (using the ORCA 5.0.3:DL_POLY 5 interface in Py‐ChemShell, see Methods) were employed to investigate the structural features and energetic properties of the two possible acyl‐enzyme tautomeric states (Figs S1 and S3). Visual inspection of QM/MM optimised geometries indicates that the *trans*‐enamine tautomer of enmetazobactam more closely resembles the crystallographically modelled enmetazobactam‐derived acyl‐enzyme than does the imine (Fig. S13). Detailed analysis of the enmetazobactam C2‐C3‐C4 and C3‐C4‐N5 angles, C2‐C3‐C4‐N5 torsion angle, and the distances between enmetazobactam N5‐HEPES O1, and between enmetazobactam N5—enmetazobactam O10, confirms that the QM/MM optimised *trans*‐enamine, rather than the QM/MM optimised imine, more closely matches the models refined into the electron density (Fig. S14, Table S5). Further, QM calculations on active site models (Fig. S4) of acylated enmetazobactam, and QM calculations of isolated acylated enmetazobactam, indicated that the *trans*‐enamine is the lower energy tautomer (Fig. S3, Table S6). Taken together, these data suggest that the crystal structure of the GES‐1:enmetazobactam complex predominantly contains the *trans*‐enamine. Subsequently, the crystal structure of the enmetazobactam‐derived acyl‐enzyme was modelled using a ring‐opened enmetazobactam‐derived covalent adduct, attached to Ser70, in the *trans*‐enamine tautomer (Fig. 3, Figs S1 and S3).

## Discussion

GES‐1 is an ESBL capable of efficiently hydrolysing a range of penicillin and cephalosporin substrates, but not carbapenems. GES‐1 is unusual among ESBLs in that it contains active site features, such as a disulphide bridge between Cys69 and Cys238, which are present in class A carbapenemases such as KPC‐2 and SFC‐1, and that are thought to be determinants of carbapenem hydrolysis [76]. Despite this, GES‐1 lacks carbapenemase activity. Nevertheless, GES‐1 possesses a combination of residues that promote or inhibit carbapenemase activity at positions proposed to configure the active site electric field in carbapenem‐derived acyl‐enzyme complexes [77]. Notably, and unusually among ESBLs, some single point mutations (G170S and G170N) convert GES‐1 into a carbapenemase [17, 18, 78]. Consequently, features in, and close to, the active site of class A β‐lactamases probably impact not only substrate turnover, but also susceptibility to inhibition by substrate‐based inhibitors in particular, as highlighted by fold differences in IC_50_ values for enmetazobactam and tazobactam inhibition of different class A β‐lactamases (Table 1).

PAS inhibitors, in particular enmetazobactam, inhibit ESBLs with greater potency than class A carbapenemases (Table 1). This is probably related to different inhibition mechanisms: enmetazobactam inhibits carbapenemases via the formation of a stable acyl‐enzyme complex that, however, turns over within 24 h, while ESBLs are inhibited by eventual irreversible active site cross link formation [37]. Here, we provide mass spectrometric, kinetic and crystallographic evidence that enmetazobactam is a moderately potent inhibitor of the ESBL GES‐1 that acts through the formation of the type of stable acyl‐enzyme observed previously in class A carbapenemases, rather than through the irreversible active site cross link formation identified previously as the mechanism of enmetazobactam inhibition of other ESBLs.

Enmetazobactam is a more potent inhibitor of GES‐1 than tazobactam *in vitro* (Table 1, Tables S1–S3), despite the close structural similarity of these two PAS inhibitors. Our data suggest that the onset of acylation (i.e. *k*_inact_/*K*) is the greatest contributor to this difference, indicating that the cationicity localised to the triazole ring of enmetazobactam may have a significant effect on binding and acylation, potentially altering the strength of interactions between enzyme and inhibitor. Although the mechanistic complexity of PAS inhibition of β‐lactamases (Scheme S1) complicates both the treatment of kinetic data and comparisons between different enzyme systems, it is of interest to compare activities across class A β‐lactamases. The greater inhibitory activity of enmetazobactam towards GES‐1, compared to tazobactam, contrasts with data for other β‐lactamases (Table 1): TEM‐26 and TEM‐116 are more strongly inhibited by tazobactam, while the two PAS inhibitors are approximately equipotent towards CTX‐M‐15 and TEM‐30. Furthermore, the IC_50_ values for both inhibitors towards GES‐1 presented here are closer to those reported previously for inhibitor‐resistant TEM and SHV variants than for other class A ESBLs and, in contrast with previous data for TEM‐116, but consistent with those for less potently inhibited enzymes such as AmpC or OXA‐10, show time dependence (Table S3). This indicates that GES enzymes may have intrinsically reduced susceptibility to mechanism‐based inhibitors, including PAS compounds and clavulanic acid (IC_50_ values for GES‐1 inhibition by clavulanic acid range between 5 and 7.7 μm [14, 15, 16]).

Although the enmetazobactam‐derived acyl‐enzyme makes few direct interactions with residues in the GES‐1 active site, bridging interactions are evident with several bound water molecules. The presence of multiple water molecules in the active site appears to enable formation of a hydrogen‐bonding network between the catalytic Glu166 and the acyl group of the covalent adduct. This network, where one water molecule also forms a hydrogen bond to the sulphone group of the enmetazobactam‐derived acyl‐adduct, may also form electrostatic interactions that enable appropriate positioning of a water molecule for the deacylation reaction that is required to complete enmetazobactam turnover (Fig. 3). Consistent with this observation, regeneration of the uncomplexed enzyme is observed by mass spectrometry after 24 h incubation of the complex (Fig. 2). The presence of a deacylating water molecule may explain why GES‐1 is inherently less susceptible to inhibition by enmetazobactam than other class A ESBLs (Table 1).

The interaction between Glu104 and the positively charged methyltriazole ring of the enmetazobactam‐derived acyl‐enzyme also may contribute to the enhanced potency of enmetazobactam, compared to tazobactam, realised through enhanced *k*_inact_/*K* values (Table S1). This favourable electrostatic interaction, not possible with the uncharged triazole ring of tazobactam, may increase the stability of the enmetazobactam acyl‐enzyme, preventing fragmentation (which is seen for the tazobactam‐derived acyl‐enzyme (Fig. 3)), and resulting in more potent inhibition, as reflected in the IC_50_ values. The presence of a HEPES molecule in the active site of the crystallised complex may provide additional electrostatic stabilisation of acylated enmetazobactam (Fig. 3). The impact of this HEPES molecule in stabilising a particular covalent adduct, or of promoting specific breakdown pathways *in crystallo*, is unknown.

Our QM/MM, active site QM and acyl‐adduct only QM models (Fig. S4), derived using DFT calculations, showed that the *trans*‐enamine is the dominant tautomeric form of acylated enmetazobactam. The magnitude of the stability difference between the *trans*‐enamine and the imine depends on the hydrogen bonding of two nearby crystallographic water molecules, according to the QM/MM calculations (Fig. S15). The proximity of these two water molecules to the enmetazobactam N5 nitrogen (Fig. S14) involved in tautomerisation, and their impact on the relative energies of the two tautomers (Table S6), suggests that they may be important for, and perhaps participate actively in, tautomerisation. The impact of including these two water molecules, that may not have been included in small active site models (Fig. S4), demonstrates the advantage of using a full enzyme QM/MM model with explicit solvent, such as that offered by the ORCA 5.3.0:DL_POLY 5 interface in Py‐ChemShell, which includes the protein and solvent environment, for modelling protein‐ligand systems. QM/MM calculations provide a practical method for modelling different chemical species in enzymes, including the vital effects of interactions with the protein, essential for investigating stability questions (and which may not be included in small models). This work also further indicates the usefulness of QM/MM geometry optimisation as part of crystallographic refinement in cases where ligand electron density is inconclusive [79, 80].

In conclusion, our work shows how enmetazobactam and tazobactam inhibit the clinically important GES‐1 β‐lactamase, and extends evidence that enmetazobactam inhibits different class A β‐lactamases in mechanistically different ways. We show that lysinoalanine cross link formation is not the sole mechanism of inhibition for class A ESBLs [37] by (enme)tazobactam and, subsequently, that reversible covalent inhibition by these compounds is not specific to class A carbapenemases. We observe that the newly approved PAS inhibitor enmetazobactam can inhibit the clinically emerging β‐lactamase, GES‐1, by formation of a 214 Da inhibitory acyl‐enzyme. This inhibition mechanism differs from our observation of the inhibition of GES‐1 by tazobactam, which (based on crystallographic observations) forms a 70 Da inhibitory complex after fragmentation of the initial bound adduct. QM calculations, at the DFT level of theory, indicate that the population of enmetazobactam‐derived 214 Da covalent adducts acylated in the GES‐1 active site is dominated by the *trans*‐enamine, rather than the imine, tautomer. Understanding the chemical details of inhibition is important in developing new inhibitors and understanding potential resistance mechanisms.

The results of this study contribute to growing evidence that GES β‐lactamases behave distinctly from other class A β‐lactamases. This family of enzymes has features characteristic of both ESBL and carbapenem‐hydrolysing enzymes, including single amino acid substitutions which improve carbapenem‐hydrolysing activity, and interactions with PAS compounds that are more similar to that of the carbapenem‐hydrolysing KPC‐2 than the ESBL CTX‐M‐15 [17, 18, 77, 81]. Therefore, there may be differences between existing, or newly emerging, GES variants in enmetazobactam susceptibility. To our knowledge, this is as yet little explored for GES‐producing clinical isolates, although a recent study that included GES‐1, GES‐2 and GES‐5 identified no variant dependence of cefepime/enmetazobactam susceptibility for recombinant *E. coli* or *P. aeruginosa* [82]. It is possible that the greater concentration of enmetazobactam than tazobactam in the bacterial periplasm may reduce the impact on bacterial susceptibility of variant‐specific differences in inhibitory potency. These considerations notwithstanding, the work presented here shows that enmetazobactam inhibits class A β‐lactamases by multiple mechanisms and suggests that strategies designed to promote fragmentation to stable covalent adducts or evade β‐lactamase‐catalysed turnover, by, for example, disrupting interactions involving the deacylating water molecule, may further enhance potency towards targets such as the GES enzymes.

## Conflict of interest

Stuart Shapiro cofounded Allecra Therapeutics. Robert A. Bonomo and James Spencer have received funding from Allecra Therapeutics.

## Author contributions

MB, PH, JS, RAB and AJM conceived the experiments. JS and AJM supervised the PhD work of MB. MB performed all wet‐lab experiments. PH and CLT supervised MB in protein purification, crystallisation and kinetic determinations. CRB and KMP‐W performed the mass spectrometry experiments. MH and MWVK advised on simulations and assisted MB in QM/MM geometry optimisation and single point energy calculations. MB, PH and MH contributed to data interpretation. MB wrote the initial manuscript draft, with contributions from SS and revisions and approval by all authors.

## Supporting information


**Scheme S1.** Kinetic scheme for inhibition of class A β‐lactamases by penicillanic acid sulphone (PAS) compounds.
**Fig. S1.** Breakdown products of penicillanic acid sulphone (PAS) inhibitors after exposure to class A β‐lactamases.
**Fig. S2.** Formation of active‐site lysinoalanine cross‐link.
**Fig. S3.** Tautomerisation of penicillanic acid sulphone (PAS) inhibitor acyl‐enzyme complexes.
**Fig. S4.** Quantum mechanics/molecular mechanics (QM/MM) set up.
**Fig. S5.** *K*_iapp_ and *k*_inact_/*K* values for Guiana extended‐spectrum (GES)‐1 inhibition by tazobactam and enmetazobactam.
**Fig. S6.** *k*_off_ values for Guiana extended‐spectrum (GES)‐1 inhibition by tazobactam and enmetazobactam.
**Fig. S7.** Omit density for Guiana extended‐spectrum (GES)‐1 bound tazobactam.
**Fig. S8.** Interactions between Guiana extended‐spectrum (GES)‐1 and penicillanic acid sulphone (PAS) inhibitor‐derived covalent adducts.
**Fig. S9.** Omit density for bound HEPES.
**Fig. S10.** Binding of intact tazobactam at crystallographic interface.
**Fig. S11.** Omit density for covalently bound enmetazobactam‐derived molecule.
**Fig. S12.** Omit density for bound HEPES in comparison with previously modelled Guiana extended‐spectrum (GES)‐2: tazobactam breakdown product.
**Fig. S13.** Models of enmetazobactam‐derived species in Guiana extended‐spectrum (GES)‐1 complex structures.
**Fig. S14.** Atom numbering of enmetazobactam and HEPES.
**Fig. S15.** Proximal water molecules affect relative stability of the trans‐enamine.
**Table S1.** Guiana extended‐spectrum (GES)‐1 inhibition kinetics with penicillanic acid sulphone (PAS) inhibitors.
**Table S2.** Guiana extended‐spectrum (GES)‐1 reactivation (*k*_off_) kinetics over time.
**Table S3.** Guiana extended‐spectrum (GES)‐1 IC_50_ values.
**Table S4.** Crystallographic statistics.
**Table S5.** Comparison of distances and bond angles in models of the enmetazobactam‐derived species.
**Table S6.** Relative energies of tautomeric species from quantum mechanics (QM) and quantum mechanics/molecular mechanics (QM/MM) calculations.

## Data Availability

Input files and ligand parameters for all QM/MM geometry optimisations and single point energy calculations are made available at the University of Bristol Research Data Repository (https://data.bris.ac.uk/). All crystal structures presented here have atomic coordinates and structure factors deposited to the Protein Data Bank (PDB; https://www.rcsb.org/) under accession codes 9ENV, 9ENW, 9ENX and 9ENY.
