# Supplementary material for: Mechanistic basis for inhibition of the extended‐spectrum β‐lactamase GES‐1 by enmetazobactam and tazobactam
Source: FEBS Lett. 2025 Sep 13;599(22):3284–300. doi: 10.1002/1873-3468.70155 (PMC12643063; doi:10.1002/1873-3468.70155)
Supplement: Supplementary file 1 — Scheme S1. Kinetic scheme for inhibition of class A β‐lactamases by penicillanic acid sulphone (PAS) compounds. Fig. S1. Breakdown products of penicillanic acid sulphone (PAS) inhibitors after exposure to class A β‐lactamases. Fig. S2. Formation of active‐site lysinoalanine cross‐link. Fig. S3. Tautomerisation of penicillanic acid sulphone (PAS) inhibitor acyl‐enzyme complexes. Fig. S4. Quantum mechanics/molecular mechanics (QM/MM) set up. Fig. S5. K iapp and k inact/K values for Guiana extended‐spectrum (GES)‐1 inhibition by tazobactam and enmetazobactam. Fig. S6. k off values for Guiana extended‐spectrum (GES)‐1 inhibition by tazobactam and enmetazobactam. Fig. S7. Omit density for Guiana extended‐spectrum (GES)‐1 bound tazobactam. Fig. S8. Interactions between Guiana extended‐spectrum (GES)‐1 and penicillanic acid sulphone (PAS) inhibitor‐derived covalent adducts. Fig. S9. Omit density for bound HEPES. Fig. S10. Binding of intact tazobactam at crystallographic interface. Fig. S11. Omit density for covalently bound enmetazobactam‐derived molecule. Fig. S12. Omit density for bound HEPES in comparison with previously modelled Guiana extended‐spectrum (GES)‐2: tazobactam breakdown product. Fig. S13. Models of enmetazobactam‐derived species in Guiana extended‐spectrum (GES)‐1 complex structures. Fig. S14. Atom numbering of enmetazobactam and HEPES. Fig. S15. Proximal water molecules affect relative stability of the trans‐enamine. Table S1. Guiana extended‐spectrum (GES)‐1 inhibition kinetics with penicillanic acid sulphone (PAS) inhibitors. Table S2. Guiana extended‐spectrum (GES)‐1 reactivation (k off) kinetics over time. Table S3. Guiana extended‐spectrum (GES)‐1 IC50 values. Table S4. Crystallographic statistics. Table S5. Comparison of distances and bond angles in models of the enmetazobactam‐derived species. Table S6. Relative energies of tautomeric species from quantum mechanics (QM) and quantum mechanics/molecular mechanics (QM/MM) calculations. [file FEB2-599-3284-s001.docx]

Supporting Information for:

Mechanistic basis for inhibition of the extended spectrum class A β-lactamase GES-1 by enmetazobactam and tazobactam

Michael Beer^a,b^, Philip Hinchliffe^a^, Marko Hanževački^b^, Christopher R. Bethel^c^, Catherine L. Tooke^d^ , Marc W. Van der Kamp^e^, Krisztina M. Papp-Wallace^c,f,g^, Robert A. Bonomo^c,f,g,h,i,j,k,l^, Stuart Shapiro^m^, Adrian J. Mulholland^b,^*,and James Spencer^a,^*

^a^ School of Cellular and Molecular Medicine, University of Bristol, Bristol, United Kingdom

^b^ Centre for Computational Chemistry, School of Chemistry, University of Bristol, Bristol, United Kingdom

^c^ Research Service, Louis Stokes Cleveland Department of Veterans Affairs, Cleveland, Ohio, USA

^d^ Department of Biology and Biochemistry, 4 South, University of Bath, United Kingdom

^e^ School of Biochemistry, University of Bristol, United Kingdom

^f^ Department of Medicine, Case Western Reserve University School of Medicine, Ohio, USA

^g^ Department of Biochemistry, Case Western Reserve University School of Medicine, Ohio, USA

^h^ Department of Molecular Biology and Microbiology, Case Western Reserve University School of Medicine, Cleveland, Ohio, USA

^i^ Department of Pharmacology, Case Western Reserve University School of Medicine, Cleveland, Ohio, USA

^j^ CWRU-Cleveland VAMC Center for Antimicrobial Resistance and Epidemiology (Case VA CARES), Cleveland, Ohio, USA

^k^ Department of Proteomics and Bioinformatics, Case Western Reserve University School of Medicine, Cleveland, Ohio, USA

^l^ Clinician Scientist Investigator, Louis Stokes Cleveland Department of Veterans Affairs, Cleveland, Ohio, USA

^m^Harry Lime Institute for Penicillin Research, Basel, Switzerland

*Address correspondence to: [adrian.mulholland@bristol.ac.uk](mailto:adrian.mulholland@bristol.ac.uk); [jim.spencer@bristol.ac.uk](mailto:jim.spencer@bristol.ac.uk)

Present Address – KMPW: JMI Laboratories, a subsidiary of Element Materials Technology, North Liberty, Iowa, USA

***Scheme S1: Kinetic Scheme for Inhibition of Class A β-Lactamases by Penicillanic Acid Sulphone (PAS) Compounds.*** *Upon formation of the Michaelis complex (E:C), PAS compounds (represented by C) can acylate the enzyme to form an initial acyl-enzyme complex (E-I). This acyl-enzyme can linearise and further fragment to form numerous covalent adducts (k_3_, represented by E-I*, see Figure S1) or deacylate (k_6_) to form the hydrolysed, non-linearised product (represented by P). The differing covalent adducts (I*) can be deacylated (k_4_), forming multiple product compounds (represented by P*). Experimentally determined values for k_off_ then include contributions from k_-1_, k_3_, k_4_, k_5_, k_6_ and k_7_. k_1_, k_-1_ and k_2_ make the major contributions to calculated values for k_inact_/K (see Figure S5, Table S1). In some class A SBLs, including Cefotaxime-Munich (CTX-M)-15, E-I* can also break down to form an irreversible active site lysinoalanine cross-link (E-LA) with release of the fragmented/linearised product (k_8_, k_9_).*

***Figure S1: Breakdown Products of Penicillanic Acid Sulphone (PAS) Inhibitors After Exposure to Class A β-Lactamases.*** *R denotes attachment of substituents differentiating sulbactam, tazobactam and enmetazobactam. The expected mass increase that would be observed by mass spectrometry experiments if each product was acylated to the enzyme is shown in brackets. 3,3-Dihydroxypropanoic acid is the (non-covalent) deacylation product of the hydrated aldehyde, and consequently is not associated with any mass increase compared to uncomplexed enzyme.*

***Figure S2: Formation of Active-Site Lysinoalanine Cross-Link.*** *The mechanism of enzyme-catalysed formation of lysinoalanine from the Ser70-(enme)tazobactam acyl-enzyme complex is unknown, but is thought to include L- to D- epimerisation of Ser70 and possible dehydroalanine formation^29, 37^.*

***Figure S3: Tautomerisation of Penicillanic Acid Sulphone (PAS) Inhibitor Acyl-Enzyme Complexes.*** *R denotes attachment of substituents differentiating sulbactam, tazobactam and enmetazobactam.*

***Figure S4: Quantum Mechanics/Molecular Mechanics (QM/MM) Set Up.*** *Representative structures for the QM/MM (A), active site model (B) and acyl-adduct model (C) geometry optimisations. Water molecules in the QM/MM starting structure are shown as cyan spheres. In (A) atoms treated by QM (Ser70 Cβ, Ser70 Oγ and enmetazobactam-derived covalent adduct) are shown in pink, whilst bound HEPES is shown in orange. For clarity, all hydrogen atoms other than those present in the non-covalent adduct are removed. In (B) asterisks denote atoms that were held fixed during geometry optimisation, to preserve crystallographic geometry. Images in the figure were generated using Schrödinger PyMol 3.0.0.*

***Figure S5: K_iapp_ and k_inact_/K values for Guiana Extended-Spectrum (GES)-1 inhibition by Tazobactam and Enmetazobactam.*** *Left, Dixon plots of reciprocals of initial rates of nitrocefin hydrolysis (1/V) by GES-1:PAS mixtures plotted against inhibitor concentration (tazobactam, blue; enmetazobactam, red). Right, plots of k_obs_ (pseudo-first-order rate constant for inactivation) against inhibitor concentration (coloured as left). Data points shown are the means of three replicates. The apparent second-order rate constant k_inact_/K is obtained from the slope of the fitted line.* *Error bars indicate the standard deviation.*

***Figure S6: k_off_ values for Guiana Extended-Spectrum (GES)-1 inhibition by Tazobactam and Enmetazobactam.*** *Progress curves representing recovery of nitrocefin hydrolysis by GES-1 after inhibitor exposure (tazobactam, blue; enmetazobactam, red), followed by jump-dilution to a final concentration of 10 nM GES-1. Enzyme was preincubated with 20 or 200 μM inhibitor for either 15 minutes (left) or 6 hours (right). Data points shown are the means of three replicates, with the fit to the k_off_ equation also shown^29, 52^. Note, the apparent linear regime observed for enmetazobactam after 6 h incubation likely reflects a heterogeneous population of possible free enzyme and different enzyme-inhibitor species whose dissociation is less well described by a single rate constant.*

**
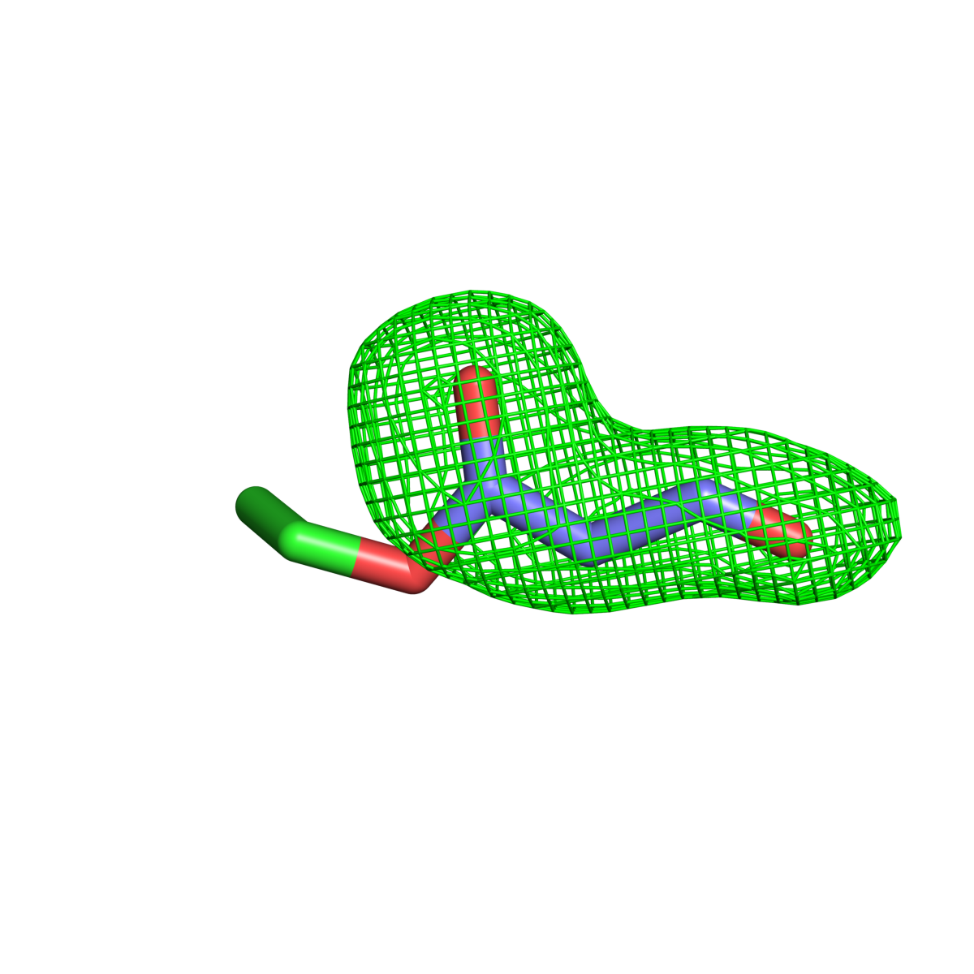
**

***Figure S7: Omit Density for Guiana Extended-Spectrum (GES)-1 Bound Tazobactam.*** *F_o_-F_c_ omit map calculated after removal of ligand is shown contoured at 3σ around atoms of the tazobactam-derived covalent adduct only. Tazobactam carbon atoms are shown blue, GES-1 (Ser70) carbon atoms in green. Images in the figure were generated using Schrödinger PyMol 3.0.0.*

***Figure S8: Interactions Between Guiana Extended-Spectrum (GES)-1 and Penicillanic Acid Sulphone (PAS) Inhibitor-Derived Covalent Adducts.*** *Interaction diagrams of tazobactam- (A) and enmetazobactam- (B) derived GES-1 acyl-enzymes. Hydrogen bonds are displayed as green dashed lines, and water molecules as cyan spheres. Images in the figure were generated using LigPlot 2.3.*

***Figure S9: Omit Density for Bound HEPES.*** *F_o_-F_c_ omit maps (green mesh) calculated after removal of ligand and contoured at 3σ around HEPES atoms in the enmetazobactam-derived (A) and tazobactam-derived (B) acyl-enzymes. Representative images shown here are taken from the active sites of chain A of both structures. Images in the figure were generated using Schrödinger PyMol 3.0.0.*

***Figure S10: Binding of Intact Tazobactam at Crystallographic Interface.*** *A) Orientation of tazobactam bound at a crystal symmetry interface (lime green chain (bottom) represents the symmetry partner of the green chain (top)). The tazobactam carboxylate moiety is within hydrogen-bonding distance of the Lys21 side chain amide. B) F_o_-F_c_ omit map (green mesh, calculated after removal of ligand) around bound tazobactam, contoured at 3σ. Images in the figure were generated using Schrödinger PyMol 3.0.0.*

***Figure S11: Omit Density for Covalently Bound Enmetazobactam-Derived Molecule.*** *F_o_-F_c_ omit map (green mesh, calculated after removal of ligand) contoured at 3σ from chain A. Density is similar in chain B. Images in the figure were generated using Schrödinger PyMol 3.0.0.*

***Figure S12: Omit Density for Bound HEPES in Comparison with Previously Modelled Guiana Extended-Spectrum (GES)-2:Tazobactam Breakdown Product.*** *F_o_-F_c_ omit map (green mesh), calculated after removal of ligand and contoured around bound HEPES in chains A of enmetazobactam-derived (A) and tazobactam-derived(B) acyl-enzymes; superposed upon previously described tazobactam-derived breakdown product observed in crystal structure of a GES-2 complex (PDB 3NIA^30^). Tazobactam-derived product was aligned to each structure separately. Alignments strongly indicate that the previously modelled compound, identified within the active site of GES-2 (GES-1 G170N point variant) after exposure to tazobactam is not a good fit to the electron density in either of the structures reported here. In both cases the F_o_-F_c_ omit map density is similar in chain B. Images in the figure were generated using Schrödinger PyMol 3.0.0.*

***Figure S13: Models of Enmetazobactam-Derived Species in Guiana Extended-Spectrum (GES)-1 Complex Structures.*** *A) Crystallographic model of trans-enamine; B) Quantum mechanics/molecular mechanics (QM/MM)-optimised trans-enamine; C) QM/MM-optimised imine. Optimised trans-enamine (B), centre) more closely resembles the structure modelled into the experimental electron density (left) than does the imine (right). Note that the methyltriazole ring (right) is poorly defined by experimental electron density, consistent with the movement of this moiety during QM/MM geometry optimisation. Images in the figure were generated using Schrödinger PyMol 3.0.0.*

***Figure S14: Atom Numbering of Enmetazobactam and HEPES.*** *Atom numbers shown are those used in the angle and distance analysis table (Table S4).*

***Figure S15****:* ***Proximal Water Molecules Affect Relative Stability of the Trans-Enamine.*** *Quantum mechanics (QM) region (enmetazobactam-derived acylated compound and Ser70 Oγ and Cβ, including bound hydrogens) is shown in magenta. Two water molecules (sticks) affect the relative stability of the trans-enamine, depending on hydrogen-bonding interactions with the Glu166 side chain and the sulphone moiety of the enmetazobactam-derived acylated compound. The lowest energy orientation (calculated using both the QM and molecular mechanics (MM) regions), compared to structures starting with different orientations of the two water molecules, is shown. QM/MM geometry optimisation were completed using the B3LYP/6-31G (d) level of theory with Grimme’s D3 dispersion correction and Becke-Johnson damping in the QM region. B3LYP-D3(BJ)/def2-TZVP was used in the QM region for single point energy calculations of the optimised structures.*

***Table S1: Guiana Extended-Spectrum (GES)-1 Inhibition Kinetics with Penicilanic Acid Sulphone (PAS) Inhibitors***

| **Inhibitor** | ***K*_iapp_ (µM)** | ***k_inact_*/*K* (M^-1^ s^-1^)** |
| --- | --- | --- |
| tazobactam | 0.55 (0.05) | 51.0 (19.8) |
| enmetazobactam | 0.48 (0.06) | 8400 (2080) |
| Standard errors are in parenthesis. | | |

***Table S2: Guiana Extended-Spectrum (GES)-1 Reactivation (k_off_) Kinetics Over Time***

|  | ***k*_off_ (s^-1^) preincubation time** | | **t_1/2_ (min)** | | ***k*_off_/ (*k*_inact_/*K*) (µM)** |
| --- | --- | --- | --- | --- | --- |
| **Inhibitor** | **15 min** | **6 hours** | **15 min** | **6 hours** | **15 min** |
| Tazobactam | 0.0013 (0.00045) | 0.0041 (0.0011) | 8.8 | 2.8 | 26.0 |
| Enmetazobactam | 0.0059  (0.00096) | 0.0074  (0.0021) | 2.0 | 1.6 | 0.70 |
| Standard errors are in parenthesis. | | | | |  |

***Table S3: Guiana Extended-Spectrum (GES)-1 IC50 Values***

|  | **IC_50_ (nM) preincubation time** | |
| --- | --- | --- |
| **Inhibitor** | **10 min** | **1 hour** |
| Tazobactam | 444 | 115 |
| Enmetazobactam | 107 | 51.7 |
|  | | |

***Table S4: Crystallographic Statistics***

|  | GES-1:enmetazobactam | GES-1:tazobactam | Uncomplexed GES-1  (enmetazobactam soaking condition) | Uncomplexed GES-1 (tazobactam soaking condition) |
| --- | --- | --- | --- | --- |
| **PDB Code** | **9ENX** | **9ENY** | **9ENW** | **9ENV** |
| **Data Collection** |  |  |  |  |
| Wavelength (Å) | 0.73379 | 0.97628 | 0.81530 | 0.7838 |
| Resolution Range | 81.04 – 1.23  (1.25-1.23) | 63.90 – 1.30  (1.32-1.30) | 52.71-1.66  (1.69-1.66) | 43.89-1.60  (1.63-1.69) |
| Space Group | *P* 21 | *P* 21 21 21 | *P* 21 | *P* 21 21 21 |
| Molecules/ASU | 2 | 2 | 2 | 2 |
| Cell Dimensions |  |  |  |  |
| a, b, c (Å) | 42.80, 81.03, 71.55 | 75.95, 80.71, 104.55 | 42.63, 80.610, 71.07 | 75.56, 80.81, 104.54 |
| α, β, γ (°) | 90, 90, 90 | 90, 90, 90 | 90, 90, 90 | 90, 90, 90 |
| Multiplicity | 6.9 (6.8) | 13.2 (13.4) | 6.8 (6.9) | 14.0 (13.9) |
| Completeness (%) | 99.8 (96.0) | 100.0 (100.0) | 100.0 (100.0) | 100.0 (99.0) |
| I/σ(I) | 8.1 (0.3) | 12.6 (0.6) | 5.4 (0.7) | 6.9 (0.4) |
| R_pim_ | 0.045 (1.151) | 0.026 (1.331) | 0.085 (1.151) | 0.064 (1.083) |
| CC_1/2_ | 0.997 (0.314) | 0.999 (0.340) | 0.995 (0.249) | 0.997 (0.324) |
| **Refinement** |  |  |  |  |
| Resolution | 70.04-1.23 | 61.42-1.36 | 41.79-1.66 | 43.89-1.60 |
| No. reflections | 135565 | 115448 | 55561 | 84864 |
| R-work/R-free | 0.1519/0.1935 | 0.1516/0.1946 | 0.2022/0.2511 | 0.1777/0.2113 |
| No. non-H atoms |  |  |  |  |
| Protein | 4096 | 4102 | 4044 | 4126 |
| Solvent | 674 | 644 | 520 | 626 |
| Ligand | 72 | 30 | - | - |
| Average B-Factors |  |  |  |  |
| Protein | 19.8 | 30.78 | 25.7 | 27.5 |
| Solvent | 35.2 | 35.6 | 32.7 | 37.9 |
| Ligand | 27.9 | 33.7 | - | - |
| R.m.s Deviations |  |  |  |  |
| Bond Lengths (Å) | 0.008 | 0.008 | 0.01 | 0.01 |
| Bond Angles (°) | 0.959 | 0.982 | 1.052 | 0.988 |
| Ramachandran (%) |  |  |  |  |
| Outliers | 0.0 | 0.0 | 0.0 | 1.6 |
| Favoured | 98.1 | 98.9 | 97.5 | 98.1 |

***Table S5: Comparison of Distances and Bond Angles in Models of the Enmetazobactam-Derived Species.*** *Measurements are shown for crystallographic model of bound ligand, quantum mechanics/molecular mechanics (QM/MM)-optimised and quantum mechanics (QM)-optimised trans-enamine and imine species. (Note that HEPES was not included in the cluster or small QM models, so no values are reported for the HEPES^O1^-N5 distance.) See Figure S14 for atom numbering.*

| **Analysis** | **X-ray**  **Modelled *Trans-*Enamine** | **X-ray**  **Modelled Imine** | **QM/MM Optimised *Trans*-Enamine** | **QM/MM Optimised Imine** | **Cluster Model *Trans*-Enamine** | **Cluster Model Imine** | **Acyl-Adduct Model *Trans*-Enamine** | **Acyl-Adduct Model Imine** |
| --- | --- | --- | --- | --- | --- | --- | --- | --- |
| C2-C3-C4 Angle (°) | 120.9 | 119.5 | 120.6 | 114.4 | 121.3 | 116.2 | 119.2 | 112.1 |
| C3-C4-N5 Angle (°) | 125.2 | 120.0 | 124.4 | 121.1 | 123.9 | 120.7 | 126.1 | 120.7 |
| C2-C3-C4-N5 Torsion Angle(°) | –172.8 | –178.2 | 177.3 | 125.8 | 173.3 | 149.3 | –178.9 | –125.7 |
| HEPES^O1^-N5 Distance (Å) | 4.7 | 4.6 | 4.6 | 4.1 | - | - | - | - |
| O10-N5 Distance (Å) | 2.7 | 2.7 | 2.9 | 3.1 | 2.9 | 2.9 | 3.0 | 2.9 |

***Table S6: Relative Energies of Tautomeric Species from quantum mechanics (QM) and quantum mechanics/molecular mechanics (QM/MM) Calculations.*** *Values for modelled imines were used as reference energies. In each case the level of theory for the geometry optimisation was B3LYP 6-31G (d) with Grimme’s D3 dispersion correction and Becke-Johnson damping whilst B3LYP-D3(BJ)/def2-TZVP was used for single point energy calculations.*

|  | **Relative Energy (kcal mol^-1^)** | | |
| --- | --- | --- | --- |
| **Tautomer State** | **QM/MM** | **Active Site Model** | **Acyl-Adduct Only** |
| Imine | 0 | 0 | 0 |
| *Trans*-Enamine | –3.3 | –9.2 | –5.7 |
